# Health education interventions to reduce cannabis and tobacco smoking-related harms among people who use cannabis: a systematic review

**DOI:** 10.1093/her/cyag016

**Published:** 2026-05-21

**Authors:** M K Nottage, K East, D Robson, T P Freeman, L Hines, R Stanbridge, H Walsh, L Brose

**Affiliations:** Nicotine Research Group, Department of Addictions, King’s College London, 4 Windsor Walk, London SE5 8AF, United Kingdom; Nicotine Research Group, Department of Addictions, King’s College London, 4 Windsor Walk, London SE5 8AF, United Kingdom; Department of Primary Care and Public Health, Brighton and Sussex Medical School, University of Brighton and University of Sussex, Brighton BN1 9PX, United Kingdom; Nicotine Research Group, Department of Addictions, King’s College London, 4 Windsor Walk, London SE5 8AF, United Kingdom; Addiction and Mental Health Group, Department of Psychology, University of Bath, Claverton Down, Bath BA2 7AY, United Kingdom; Addiction and Mental Health Group, Department of Psychology, University of Bath, Claverton Down, Bath BA2 7AY, United Kingdom; Addiction and Mental Health Group, Department of Psychology, University of Bath, Claverton Down, Bath BA2 7AY, United Kingdom; Nicotine Research Group, Department of Addictions, King’s College London, 4 Windsor Walk, London SE5 8AF, United Kingdom; Addiction and Mental Health Group, Department of Psychology, University of Bath, Claverton Down, Bath BA2 7AY, United Kingdom

## Abstract

Cannabis is commonly smoked and often co-used with tobacco, yet evidence on interventions to reduce smoking harms for people who use cannabis is limited. We systematically reviewed evidence on the efficacy and effectiveness of health education interventions (aiming to increase health literacy, knowledge, or motivation) in reducing cannabis smoking and tobacco smoking among people using cannabis. We searched five databases (inception–February 2025) for quantitative evaluations of health education interventions targeting cannabis use and reporting cannabis and/or tobacco smoking among people who use cannabis. Evidence from 32 studies was synthesized narratively, with risk of bias assessed using design-appropriate tools. Samples ranged from 15 to 10 781 participants, predominantly adolescents and young adults. Half evaluated interventions to prevent uptake, mostly school-based programmes, which showed mixed impact in reducing cannabis smoking. Half promoted quitting/reduction and provided some evidence that motivational interventions reduced cannabis smoking. Tobacco outcomes were rarely assessed (*k* = 7) and largely null; risk of bias was high throughout. Overall, school-based preventive programmes showed mixed effects, while motivational interventions more consistently reduced cannabis but not tobacco smoking. Even modest effects may benefit public health, highlighting the need to clarify effective components, delivery settings, and to standardize measures for routes of administration and co-use.

## Introduction

With an estimated 244 million people reporting past-year use in 2023, cannabis is one of the most used substances worldwide [1]. Global use increased by 28% between 2012 and 2022, a trend likely to continue as more countries legalize cannabis [1, 2]. Considering this high prevalence, even modest risks associated with use can have substantial public health implications. In 2019, cannabis use disorders accounted for 41% of all drug use disorder cases globally [1]; use is linked to increased risks of motor vehicle accidents, low birth weight (maternal use), and the development of psychoses [3]. When smoked, cannabis poses risks to respiratory health, including worsening respiratory symptoms, chronic bronchitis, susceptibility to respiratory infections, and poor asthma control [3, 4]. Cannabis can be used through various routes of administration, including non-smoking routes like vapourizers and edibles; these increasingly popular routes may confer varying degrees of risk reduction compared to smoking [5–7]. Nonetheless, smoking (e.g. joints and waterpipes) is a common route of cannabis administration globally, and may remain especially popular in regions where cannabis is not legal and alternative products are less available [5, 8, 9].

Many people who use cannabis are also exposed to tobacco-related harms. In Canada, England, and the US, close to 50% of youth aged 16–19 who used cannabis in the past month reported ‘smoking cannabis with tobacco in a joint or blunt’ in 2017 [9]. Mixing and using both products together, a form of co-use referred to as co-administration, is especially prevalent in some regions like Europe [10, 11]. Also prevalent is concurrent co-use, where both products are used within a set timeframe, either separately or shortly after one another (e.g. ‘chasing’ a joint with a cigarette) [11]. Not only is tobacco smoking a leading cause of preventable illness and mortality, causing numerous serious health conditions [12], but evidence also suggests that smoking cannabis and tobacco together leads to greater toxicant exposure than smoking either alone [13, 14]. Further, co-use is associated with higher odds of problematic cannabis use (i.e. leading to impairment or distress, including cannabis use disorder) and poorer cannabis cessation outcomes compared to single-substance use [15, 16].

Consideration should be given to individuals with mental health disorders, as they are more likely to use tobacco and cannabis than people without [17–19]. Further, the co-use of cannabis and tobacco is linked to higher rates of mental health disorders than using either substance alone [20, 21]. Both tobacco and cannabis may play a causal role in the development of mental health disorders [22–25].

Smoking-related harms among people who use cannabis should be a focus for health promotion. Health education is defined by the World Health Organization (WHO) as ‘any combination of learning experiences designed to help individuals and communities improve their health by increasing knowledge, influencing motivation, and improving health literacy’ [26, 27]. It can play a critical role in behaviour change through modifying motivation and psychological capability [28]. Health education interventions encompass a broad range of approaches at individual and population levels, including mass media campaigns, product labelling, school education programmes, and brief psychoeducational or motivational interventions. These can be applied to prevent drug uptake as well as encourage behaviour change among people who use (i.e. cessation and/or harm reduction). Some have demonstrated effectiveness in addressing cannabis use; for instance, health warnings on cannabis products increase health literacy and decrease appeal [29], and both universal and targeted school-based interventions can help to prevent and reduce cannabis use [30, 31].

However, many health education interventions are abstinence-focused, and few feature content specific to cannabis smoking or the co-use of cannabis with tobacco/nicotine. While abstinence is the most effective way to prevent smoking-related harms, harm reduction can support individuals who may not quit immediately by promoting lower-risk consumption methods. In Canada, lower-risk cannabis use guidelines provide harm reduction approaches (e.g. avoiding combustible routes of administration), and harm reduction resources are increasingly available for individuals, educators, and communities [7, 32]. Such interventions are still sparse, and few have been evaluated.

### Study aims

Considering the health risks associated with smoking cannabis, especially when co-used with tobacco, identifying effective health education approaches to help people who use cannabis avoid smoking-related harms is key. Research in this area is sparse and has not yet been synthesized. The primary aim of this review is to examine the efficacy and effectiveness of health education interventions in preventing cannabis smoking behaviours, as well as reducing tobacco smoking behaviours among people who use cannabis. A secondary aim was to summarize mental health outcomes if reported, as people with mental health disorders are at higher risk for smoking-related harms.

## Materials and methods

This systematic review was registered on PROSPERO (CRD42024512945) [33]. It follows the Preferred Reporting Items for Systematic Reviews and Meta-Analyses (PRISMA) guidelines (Appendix A) [34]. Additional materials are available on the Open Science Framework (OSF) [35]. Throughout this manuscript, ‘*k*’ refers to the number of included studies.

### Search

We searched for reports published from inception to 14 February 2025, using keywords relating to cannabis, smoking, and health education, across five databases: Scopus, Embase (Ovid), Medline, Cumulative Index to Nursing and Allied Health Literature (CINAHL), and the International Bibliography of the Social Sciences (IBSS). Search strategies are provided in Appendix B and OSF [35].

### Eligibility

Eligible reports were peer-reviewed quantitative studies, excluding reviews, published in English, French, or Spanish (languages spoken by the study team). Reports were excluded if the full text could not be located and authors could not be contacted or did not respond (*k* = 10). Populations included humans of any age. Eligible interventions included health education as defined by the WHO (i.e. focused on increasing knowledge, motivation, and/or health literacy). Interventions had to include content on cannabis and/or tobacco use, whether standalone or part of broader substance use programmes.

Primary outcomes included cannabis smoking behaviour (via explicitly smoked routes of administration) and/or tobacco smoking among people who use cannabis. No restrictions were placed on frequency of cannabis use, to accommodate both prevention and cessation studies. Secondary outcomes included mental health outcomes if reported in studies measuring primary outcomes. Eligibility details are provided in Appendix B.

### Screening

Reports identified in the searches were uploaded to Rayyan, an online referencing software [36]; Rayyan’s automation tool removed duplicates with a similarity score ≥95%, those <95% were manually reviewed. Reports were screened based on titles and abstracts by one researcher (M.N.) and 20% (*k* = 2441) were independently second-screened by others (K.E., L.B., R.S., H.W., and D.R.), resulting in 96.4% agreement. Reports, where decisions differed or with insufficient information, were moved to the full-text stage. Full-text reports were retrieved online and through KCL’s library services. If eligibility was unclear, the study authors were contacted, and reports were included if sufficient information was provided. All full-text reports were screened by one researcher (M.N.) and 40% (*k* = 210) independently second-screened by another (K.E., L.B., R.S., H.W., and D.R.). This resulted in 67 conflicts (65 related to outcome measures, 5 to intervention type) and 24 cases where at least one screener selected ‘maybe’. Following discussions, all conflicts were excluded and 6 of the ‘maybe’ cases were included.

### Data extraction

A data extraction form was developed using Microsoft Excel, with categories covering study metadata and overview, sample characteristics, intervention characteristics, outcomes measures, and results. Specific items are provided in Appendix C and the full dataset on OSF [35]. For all eligible reports: (i) one researcher (M.N.) extracted all data and (ii) data on outcome measures and results were independently extracted by a second researcher (K.E., L.B., R.S., H.W., D.R., and T.F.). Differences were resolved through team discussion.

### Data synthesis

As anticipated [33], there was a high heterogeneity in study designs, intervention types, outcome measures, and analyses. Therefore, narrative synthesis was used, including effect sizes where available. Results were divided into two sections based on the stated primary aim of the interventions they evaluated: (i) preventing uptake or (ii) promoting quitting or harm reduction. Characteristics and outcomes of individual studies were synthesized according to cannabis smoking outcomes and tobacco smoking and co-use outcomes (non-exclusive; studies could report multiple outcomes). Findings on mental health outcomes were limited and therefore narratively synthesized in the Supplementary data.

### Risk of bias assessment

Risk of bias was assessed using: (i) the revised Cochrane risk of bias tool (RoB2) for randomized controlled trials; (ii) the Cochrane Risk of Bias in Non-randomized Studies–of Interventions (ROBINS-I) tool for non-randomized studies, version 2; the Newcastle-Ottawa Quality Assessment Scale: Cohort Studies (NOS Cohort) for cohort studies. One researcher (M.N.) assessed risk of bias and ambiguities were discussed among the research team.

### Deviations from protocol

Eligibility criteria were refined for consistent screening by clarifying that cannabis smoking had to be explicitly measured and that tobacco outcomes were only included when measured within cannabis-using samples. We used the NOS Cohort risk of bias assessment tool for cohort studies instead of the pre-registered tool. Details for these deviations are specified in Appendix B.

## Results

### Study selection

**Figure 1:**
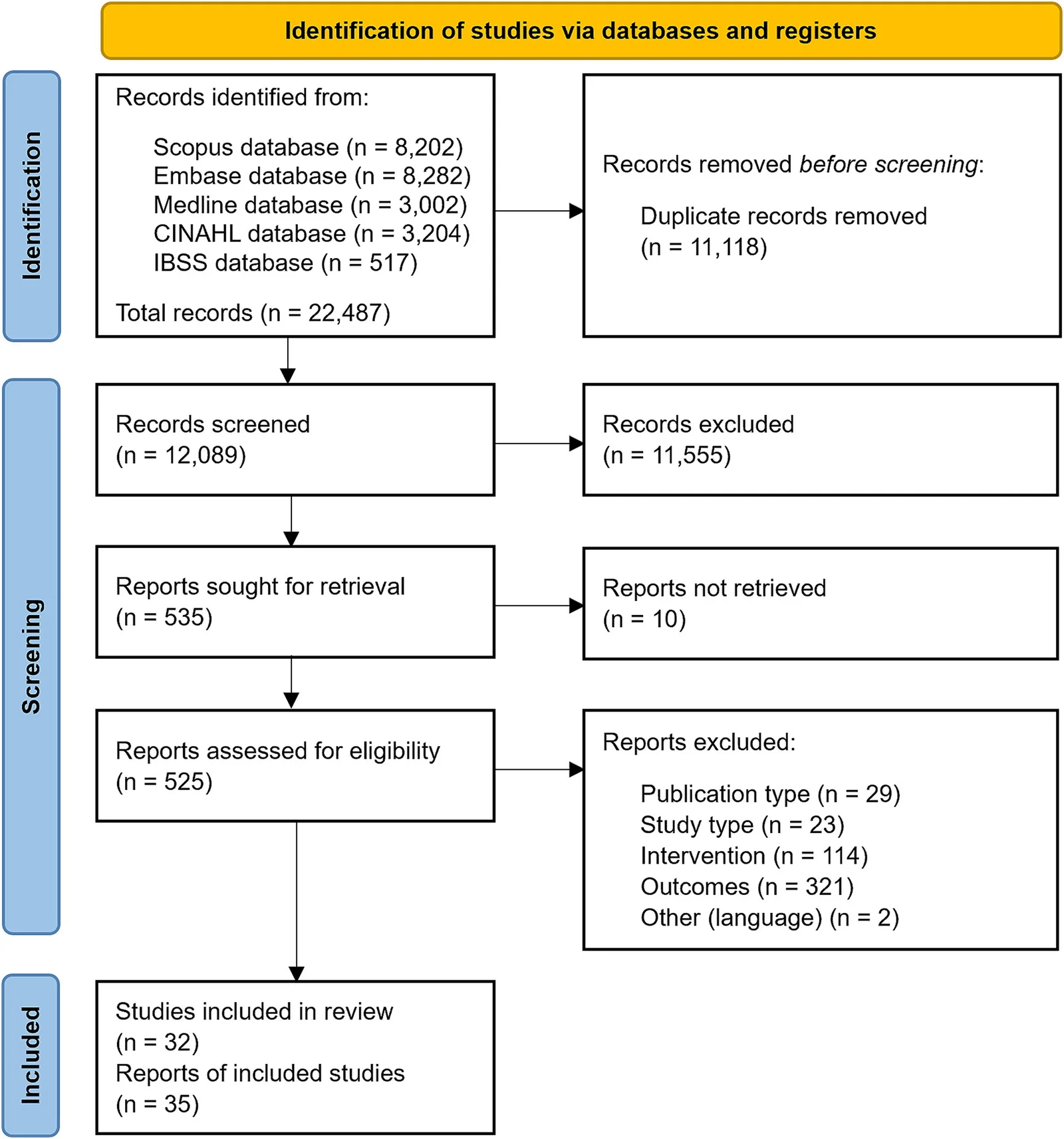
Study selection: PRISMA flow diagram.

Figure 1 shows the screening process: 12 089 reports were screened, of which 35 were included.

### Study characteristics

Table 1 summarizes study characteristics, further detailed in Tables 2 and 3. Three of the 35 reports were follow-ups [37–39], yielding 32 distinct studies. Most (78.1%) were randomized controlled trials (RCTs), conducted in the US (53.1%), and published in the 2010s (51.4%; range 1992–2023). Based on setting, providers, population, and intervention criteria [40], most studies (*k* = 21) were primarily effectiveness trials (including 5 pilot studies), while 11 were primarily efficacy trials (including 3 pilot studies). Sixteen evaluated interventions to prevent cannabis uptake (Table 2), and 16 interventions to promote cannabis cessation and/or harm reduction strategies (Table 3).

**Table 1:** Summary of the main characteristics of the 32 included studies (from 35 reports).

**Characteristic**	**Category**	**Number (%)** ^a^	**References**
**Country**	United States	17 (53.1)	[37, 38, 41–57]
	Mexico	3 (9.4)	[58–60]
	Switzerland	3 (9.4)	[61–63]
	United Kingdom	3 (9.4)	[64–66]
	Netherlands	2 (6.3)	[67, 68]
	Canada	1 (3.1)	[39, 69]
	France	1 (3.1)	[70]
	South Africa	1 (3.1)	[71]
	Uruguay	1 (3.1)	[72]
**Study design**	Randomized controlled trials (RCTs)	25 (78.1)	[37–39, 41–43, 45–50, 52–54, 57–60, 62–64, 66, 67, 69–72]
	*Of which: cluster-RCTs*	14 (43.8)	[37, 38, 45–47, 50, 52–54, 58–60, 64, 70–72]
	Cohort studies	3 (9.4)	[44, 56, 61]
	Non-randomized studies (NRS)	4 (12.5)	[51, 55, 65, 68]
**Decade published^a^^,^^b^**	2010–2019	18 (51.4)	[37, 39, 43, 44, 46, 53, 56–58, 61–64, 67–70, 72]
	2020–2025	6 (17.1)	[42, 48, 49, 59, 60, 71]
	2000–2009	8 (22.9)	[38, 41, 45, 51, 52, 54, 65, 66]
	1990–1999	3 (8.6)	[47, 50, 55]
**Intervention primary aim**	Preventing uptake	16 (50.0)	[37, 38, 45–47, 50–56, 58–60, 64, 71, 72]
	Promoting cessation or harm reduction	16 (50.0)	[39, 41–44, 48, 49, 57, 61–63, 65–70]
**Intervention type^a^**	Youth education programmes	14 (37.8)	[37, 38, 45–47, 50–55, 58–60, 71, 72]
	Motivational interventions	9 (24.3)	[41, 43, 44, 61–66]
	Psychoeducation	4 (10.8)	[62, 64, 66, 67]
	Mix of feedback, motivational, and psychoeducation techniques	5 (13.5)	[39, 42, 67–70]
	Personalized feedback	3 (8.1)	[48, 57, 62]
	Social media groups	1 (2.7)	[49]
	Television ads	1 (2.7)	[56]
**Target age group**	Adolescents (age range 10–19)	19 (59.4)	[37, 38, 43–47, 50–56, 58–60, 64–66, 72]
	Young adults (18–35)	5 (15.6)	[39, 48, 49, 57, 63, 69]
	Adolescents and young adults (13–25)	4 (12.5)	[67, 68, 70, 71]
	Adults (18+, no upper age limit)	4 (12.5)	[41, 42, 61, 62]
**Sex/gender distribution**	Balanced (within 5% of 50%)	17 (53.1)	[38, 42, 46, 47, 50–54, 56–58, 60, 64, 65, 71, 72]
	Majority male (≥55% male)	12 (37.5)	[39, 41, 43, 44, 55, 61–63, 66–70]
	Majority female (≥55% female)	2 (6.3)	[48, 49]
	Not reported	1 (3.1)	[37, 45]
**Outcome(s) measured^c^**	Cannabis smoking	28 (87.5)	[37–39, 41, 43, 45–49, 51–61, 63–72]
	Tobacco smoking	6 (18.8)	[42, 44, 62, 66, 68, 70]
	Cannabis and tobacco co-use	1 (3.1)	[50]
**Data collection**	Self-report	32 (100)	[37–39, 41–72]

a Percentages are based on the 32 included studies, except for ‘Decade published’ (based on 35 included reports) and ‘Intervention type’ (based on 37 interventions as some studies evaluated multiple).

b Searches were conducted from inception but no earlier eligible studies were identified.

c Some studies reported more than one outcome.

**Table 2:** Studies evaluating uptake-prevention health education interventions which reported cannabis smoking outcomes (*k* = 15) and tobacco smoking outcomes (*k* = 1).

**Reference, country**	**Design,** **conditions,** **waves (last follow-up), setting**	**Sample size, age, setting, sample characteristics**	**Baseline cannabis, tobacco, mental health**	**Intervention(s) format and content, comparator**	**Outcome measure(s), analysis adjusted for**	**Intervention effect(s) at last follow-up (unless specified)**	**Risk of bias (Tool)**
*Cannabis smoking outcomes*							
**Alvaro, 2013,** **USA [56]**	**Cohort** (effectiveness), 1 condition, 2 waves (12 months), Unspecified setting	*N* = 2993 adolescents aged 12–18, 50.79% male, 67.02% White, 15.6% AA, 13.46% Hispanic, 3.91% Asian	12.8% ever cannabis use	**Television ads**: Participants viewed 1 to 5 videos from the ‘National Youth Anti-drug Media Campaign’ discouraging cannabis use and/or substance use	Ever cannabis smoking, and time since last smoked. Adjusted for attitudes towards cannabis, age, gender, ads viewed	×/✓ Positive ad evaluations were associated with reduced cannabis use at follow-up (*b* = −0.031, *P* < .05). This effect was moderated by baseline use status, with significant reductions observed only among those who used (*b* = −0.111, *P* < .01), while associations were not significant for those who did not	Low risk (NOS Cohort)
**Ayers, 2023, Mexico [60]**	**Cluster RCT** (effectiveness), 3 conditions, 4 waves (26 months), Secondary school	*N* = 5522 adolescents aged 11–17, 51% male	Cannabis mean frequency = .05, SE = .01; cigarette mean frequency = .16, SE = .02 (from 0 = never to 6 = 40+ times)	**Youth education programme**: 10–12 weekly teacher-delivered sessions. Two versions of the Keepin’ it REAL curriculum (Spanish with/without cultural adaptations). Content focuses on the development of skills and knowledge to refuse substance use **Comparator:** no intervention control	Past 30-day cannabis smoking. Adjusted for stratification by urban area and clustering by school	× There were no significant direct effects of either intervention on reducing cannabis smoking	Some concerns (RoB2)
**Clayton, 1996, USA [47]**	**Cluster RCT** (effectiveness), 2 conditions, 6 waves (4 years), Secondary school	*N* = 2071 adolescents (90% aged 11–12), 50.63% male, 75% White, 22% AA	4% ever cannabis use, 28% ever cigarette use	**Youth education programme**: 16 sessions, hour-long and weekly, delivered by trained uniformed police. Drug Abuse Resistance Education (DARE) curriculum: drug education, decision-making and peer-pressure resistance skills, social norms **Comparator:** TAU control (other unspecified drug education)	Number of times of cannabis smoking in the past year. Adjusted for baseline differences within and between schools	× There were no significant differences between intervention schools and comparison schools in cannabis smoking behaviours	Some concerns (RoB2)
**Hecht, 2006, USA [51]**	**Non randomized study** (effectiveness), 4 conditions, 4 waves (14 months), Secondary school	*N* = 6298 adolescents, 47% female, 65% MA, 11% other Hispanic, 24% White, 12% AA, 11% NA, 3% Asian or PI	Not reported	**Youth education programme**: 10 teacher-delivered sessions. Three versions of the Keepin’ it REAL curriculum targeting different demographics (MA, White and AA, Multicultural). Content focuses on the development of skills and knowledge to refuse substance use **Comparator:** TAU control (schools delivering various drug education)	Past 30-day cannabis smoking, combining days smoked and number of ‘hits’ Adjusted for baseline use	✓ Combined, the interventions significantly slowed the increase in cannabis smoking over time (*b* = −0.030, SE = 0.011, *P* = .010). Separating conditions, effects were driven by the multicultural curriculum (b = −.033 (SE = .014), p = .020), while the other two curricula were non-significant	Serious risk (ROBINS-I)
**Khuzwayo, 2020, South Africa [71] ^a^**	**Cluster RCT** (effectiveness), 2 conditions, 2 waves (4 months), Secondary school	*N* = 1 558 adolescents and young adults (19% aged 13–15, 49% 16–17, 32% 18–23 in the intervention; 32% aged 13–15, 43% 16–17, 25% 18–13 in the control), 50.3% female	16% past 30-day cannabis use in the intervention (15% in control). 29% past 30-day cigarette smoking in the intervention (27% in control)	**Youth education programme:** 13 twice-weekly group sessions (45 to 60 minutes), delivered over 2 months by youth facilitators. Risk reduction intervention comprising topics on alcohol and drug abuse, peer pressure, and decision making **Comparator:** no intervention control	Cannabis smoking amount (average number of times per day). Adjusted for school and baseline characteristics	× Decrease in cannabis smoking outcomes conditions at 4-months follow-up did not differ between students who received the intervention and those in the control group (10% and 11% decrease respectively, OR = 0.9[0.6–1.3])	Some concerns (RoB2)
**Kulis, 2021, Mexico [59]**	**Cluster RCT** (effectiveness), 2 conditions, 2 waves (7 months), Secondary school	*N* = 1418 adolescents (93% aged 11–12), 49.2% female	1.9% past 30-day cannabis use, 4.8% past 30-day cigarette smoking	**Youth education programme**: 12 weekly teacher-delivered sessions. Keepin’ it REAL curriculum, culturally adapted for MA, AA, and EA cultures. Content focuses on the development of skills and knowledge to refuse substance use **Comparator:** TAU control	Past 30-day cannabis smoking (frequency and amount of joints) Adjusted for gender, age, living situation	×/✓ The intervention effect on reducing cannabis smoking compared to control was very small overall (*d* = 0.026 for frequency, −0.032 for amount), but stronger among male participants (effect sizes not reported for males)	Some concerns (RoB2)
**Kulis, 2007, USA [52]**	**Cluster RCT** (effectiveness), 2 conditions, 3 waves (6–8 months), Secondary school	*N* = 3605 adolescents, 49.1% female, 73% Hispanic, 13% White, 8% AA, 5% NA, 1% Asian	12% past 30-day cannabis smoking, 11% past 30-day cigarette smoking	**Youth education programme**: 10 sessions (complemented by student-made videos, a TV and radio 'public service announcement campaign'). Keepin’ it REAL curriculum culturally adapted for MA, AA, and EA cultures. Content focuses on the development of skills and knowledge to refuse substance use **Comparator:** TAU control (other unspecified drug education)	Past 30-day cannabis smoking (number of days) Adjusted for baseline use and English reading ability	× There were no intervention effects on reducing cannabis smoking compared to control, including when testing for gender interactions, and when comparing Hispanic and non-Hispanic groups	Some concerns (RoB2)
**Marsiglia, 2011, USA [53]**	**Cluster RCT** (effectiveness), 4 conditions, 6 waves (3.5 years), Elementary and secondary school	*N* = 783 adolescents (93% aged 10–11), 50.4% female, 84% MA	Cannabis smoking mean = 0.036, SD = 0.212; cigarette smoking mean = 0.043, SD = 0.224 (past 30-day frequency from 0 = never use to 7 = 40+ times)	**Youth education programme**: 10–12 teacher-delivered sessions over 2–4 months. Keepin’ it REAL curriculum, adapted for elementary school, delivered in elementary or secondary school or both. Content focuses on the development of skills and knowledge to refuse substance use **Comparator:** TAU control (other unspecified drug education)	Past 30-day cannabis smoking (number of times) Adjusted for waves and schools	×/✓ Compared to controls, the intervention slowed the increase in cannabis use over time for students who received it in both elementary and secondary school (*b* = −0.015, *P* < .001) or in secondary school only (*b* = −0.017, *p* < .01), but not for those who received it in elementary school only. Overall, cannabis use increased over time (*b* = 0.081, *P* < .001)	Some concerns (RoB2)
**Marsiglia, 2015, Mexico [58]**	**Cluster RCT** (effectiveness), 2 conditions, 3 waves (8 months), Secondary school	*N* = 431 adolescents (aged 12–15, M = 13.01, SD = 0.44), 45.2% male	Past 30-day cannabis 'hits' mean = 0.028, SD = 0.18 (from 0 = none to 6 = 40+). Past 30-day cigarettes mean = 0.24, SD = 0.80 (from 0 = none to 7 = 1–5 packs)	**Youth education programme**: Two teacher-delivered sessions/week. Keepin’ it REAL curriculum, in Spanish. Content focuses on the development of skills and knowledge to refuse substance use **Comparator:** No intervention control	Past 30-day cannabis smoking (number of times and number of ‘hits’) Adjusted for clustering	×/✓ The intervention had no significant overall effect on cannabis use, but a tentative gender-by-treatment interaction suggested that males in the intervention group showed less growth in use than controls (frequency: *b* = −0.070, *P* < .10; amount: *b* = −0.087, *P* < .05)	Some concerns (RoB2)
**Marsiglia, 2018, Uruguay [72]**	**Cluster RCT** (effectiveness/pilot), 2 conditions, 2 waves, Secondary school	*N* = 316 adolescents (M = 12.39, SD = 0.63), 49.7% male (age and gender data concern the intervention group only)	Cannabis mean frequency = 0.10, SD = 0.69; cigarette mean frequency = 0.05, SD = 0.21 (both past 30-day, from 0 = never to 6 = 30+ times; intervention group only)	**Youth education programme**: Ten 45-minute teacher-delivered sessions. Keepin’ it REAL curriculum, in Spanish. Content focuses on the development of skills and knowledge to refuse substance use **Comparator:** No intervention control	Past 30-day cannabis smoking (number of ‘puffs’ taken) Adjusted for baseline use	✓ The intervention significantly reduced cannabis smoking with a small-to-medium effect size (*d* = 0.32), compared to the control group which showed an increase over time	Some concerns (RoB2)
**McCambridge, 2011, UK [64]^a^**	**Cluster RCT** (effectiveness), 2 conditions, 3 waves (12 months), Further education colleges	*N* = 416 adolescents (aged 16–19, mean 17.6, SD .8), C1: 55% male, C2: 52% male, 25.49% White, 47.01% Black, 18.01% Asian, 9.5% Mixed/other	21.5% smoked cannabis (C1 = 20%, C2 = 23%), 28% smoked cigarettes (C1 = 32%, C2 = 24%)	Both conditions involved an hour-long session delivered by research staff. **C1: Motivational intervention:** MI involving imagining/discussing a series of hypothetical situations in which they might find it difficult to refuse offers of drugs they had not previously used, reasons for not using substances, and how initiation of use might affect plans. **C2: Psychoeducation:** presented as an active control, this ‘drug awareness’ condition comprised a 16-question quiz on the effects of cigarette smoking, alcohol consumption and cannabis use; discussion components; leaflets with information on the effects of target drugs; delivered while avoiding Mi techniques	Joints smoked per week Adjusted for clustering, baseline use, practitioner	×/✓ The mean number of joints smoked in the previous week significantly decreased at 3 months (M = 8.0, SD = 10.3; *P* < .01) and 12 months (M = 6.2, SD = 9.0; *P* < .001) among those who used at baseline. There was no statistically significant difference between conditions	Some concerns (RoB2)
**Okamoto, 2019, USA [46]**	**Cluster RCT** (efficacy), 4 conditions, 6 waves (up to 15 months), Secondary school	*N* = 486 adolescents (5.7% aged 10, 50.2% 11, 39.7% 12, 4.3% 13+), 52.1% female, 25.4% Chinese, 45.3% Filipino, 54.3% Hawaiian, 15.0% Hispanic, 24.3% Japanese, 5.4% Korean, 29.7% Portuguese, 7.5% Samoan, 31.0% White, 5.0% Other Asian, 12.3% Other PI	2.1% used cannabis, 4.3% used cigarettes or e-cigarettes	**Youth education programme**: 9 teacher-delivered 45-60 min sessions. Hoʻouna Pono curriculum focused on the development of skills and knowledge to refuse substance use, including videos of realistic situations culturally adapted for Hawai’i **Comparator:** Delayed intervention control	Past 30-day frequency of cannabis smoking. Adjusted for clustering and baseline differences (age and SES)	× The intervention showed no significant effect on cannabis smoking growth over time, as cohorts exposed to the programme earlier did not differ from those exposed later in either linear or quadratic growth models	High risk (RoB2)
**Spoth, 2007, USA [45] Spoth, 2011, USA [37]**	**Cluster RCT** (effectiveness), 2 conditions, 5 waves (18 months and 4.5 years), Secondary school	*N* = 10 781 adolescents (US 6th grade), *n* = 8655 completed 5th wave	Not reported	**Youth education programme**: Strengthening Families Program (SFP; 7 sessions involving parents and youth, enhancing parental skills in communication and limit-setting, and youth skills in peer resistance). Followed 11 to 15 sessions for youth: either Life Skills Training (LST), Project Alert, or All Stars (all teach substance use refusal skills and knowledge, with Alert and All Stars also focusing on peer norms) **Comparator:** Control	Ever cannabis smoking, measured as rates of people starting to use between baseline and follow-up. Adjusted for state, cohort, block, risk status, parenting practices	✓ Rates of people starting to use cannabis were significantly lower for intervention group compared to control at 18 months, with moderate to large effect sizes (community *d* = 0.62, individual *d* = 0.51). These results were maintained at the 4.5-year follow-up (community *d* = 0.54, individual *d* = 0.77)	Some concerns (RoB2)
**Spoth, 2002, USA [54] Spoth, 2008, USA [38]**	**Cluster RCT** (effectiveness), 3 conditions, 7 waves (1 year and 5.5 years), Secondary school	*N* = 1 372 adolescents (US 7th grade), *n* = 1237 completed 7th wave 53.4% Male, 96.2% White	Ever cannabis use: C1 3.5%, C2 3.0%, control 2.1%. Ever cigarette smoking: C1 25.2%, C2 26.9%, control 17.0%.	**Youth education programmes**: Compared **C1**: LST alone (15 sessions and 5 boosters, for youth) to **C2**: LST + SFP (additional 7 sessions and 4 boosters for parents and youth); see Spoth 2007 for content **Comparator:** Control	Ever cannabis smoking, measured as rates of people starting to use between baseline and follow-up Adjusted for baseline use	✓ Rates of people starting to use cannabis were significantly lower for both intervention conditions compared to control at 1 year (C1: 4.3%, C2: 4.1%, Control: 7.9%; both *P* < .05), and there were no significant differences between conditions. At 5.5-years, effects were maintained, with significantly lower cannabis initiation in intervention schools by 12th grade (C1/C2: 29.3%, Control: 38.1%; both *P* < .05)	Some concerns (RoB2)
**St Pierre, 1992, USA [55]**	**Non randomized study** (effectiveness), 3 conditions, 4 waves (27 months), Youth clubs	*N* = 161 adolescents (age M = 13.6), 75% male, 45% White, 42% AA, 14% Hispanic	Not reported	**Youth education programme**: 12 prevention staff delivered sessions, plus a second conditions with 5 boosters at 1 year and 3 boosters at 2 years. Stay SMART intervention adapted from LST, based on personal and social competence approach, including modules on cannabis **Comparator:** Control	Cannabis smoking behaviour scale, combining a measure of cannabis smoking frequency and a measure of intent to continue smoking cannabis Adjusted for baseline differences	✓ Both intervention conditions were associated with reduced cannabis smoking behaviour compared to control across the 27 months (F(2,148) = 3.34, *P* < .05)	Serious risk (ROBINS-I)
*Tobacco smoking outcomes*							
**Botvin, 1995, USA [50]**	**Cluster RCT** (effectiveness), 3 conditions, 1 wave (at 6 years), Secondary school	*N* = 3 597 adolescents, (*N* = 2 752 high-fidelity sample^b^), mean age 18.05, 52% male, 91% White	1% used cannabis monthly and 7% smoked cigarettes monthly	**Youth education programme**: 30 in-person sessions, teacher-delivered, over three years. Two conditions: teachers received an in-person training workshop (E1) versus video training (E2). Topics: information and skills for resisting social influences to use drugs, generic personal and social skills for increasing overall competence **Comparator:** TAU control	Co-use outcome combining frequency of cannabis use (mode not specified) and cigarette smoking. Adjusted for baseline use	✓ Weekly cannabis and cigarette co-use decreased significantly in the general (E1 = 0.04, E2 = 0.04 versus C = 0.08, *P* < .05) and high-fidelity samples (E1 = 0.02, E2 = 0.03, C = 0.08, *P* < .05). Monthly co-use declined only in the high-fidelity sample (E1 = 0.05, E2 = 0.07, C = 0.12, *P* < .05)	Some concerns (RoB2)

a These interventions primarily aimed to prevent uptake but did contain notable treatment and harm reduction components.

b The high-fidelity sample in Botvin *et al.* [48] refers to a subsample of individuals who received at least 60% of the intervention.

Symbols: ✓ significant reduction, × no significant reduction, and ×/✓ mixed findings. AA, African American; CBT, cognitive behavioural therapy; MA, Mexican American; MI, motivational interviewing; NA, Native American; PI, Pacific Islander; RCT, randomized controlled trial; TAU, treatment as usual.

#### Sample characteristics

Sample sizes ranged from 15 to 10 781. Most studies (59.4%) included adolescents (ages 10–19), young adults (15.6%; ages 18–35), or both (12.5%), with four studies including adults (12.5%, ages 18+ with no upper limit) [41, 42, 61, 62]. All but one uptake prevention study included adolescents. While 17 studies (53.1%) had approximately equal male/female participation, 12 (37.5%) were male dominated; among interventions to promote cessation and/or harm reduction, males comprised over 80% of participants in six studies [43, 44, 61–63, 67]. Racial/ethnic composition varied where reported; 11 studies omitted these data [37, 43, 56–58, 59, 61, 65, 66, 69, 70, 71].

Among interventions to prevent uptake, baseline cannabis use was lowest among younger samples, e.g. 2%–4% in 10- to 12-year-olds [46, 47, 59] versus 16%–21.5% in older adolescents/young adults [64, 71]. Most cessation/harm reduction studies required cannabis use for inclusion, ranging from occasional (e.g. ≥1 joint/month for a year [70]), to heavy (e.g. DSM-IV dependence, use on ≥40/90 days [41]). Four studies reported baseline rates of anxiety, depression, or psychotropic medication use [42, 48, 49, 70]. Two studies required a psychotic disorder diagnosis for inclusion [61, 63].

**Table 3:** Studies evaluating cessation and/or harm reduction focused health education interventions which reported cannabis smoking outcomes (*k* = 13) and tobacco smoking outcomes (*k* = 6).

**Reference, country**	**Design,** **conditions,** **waves (last follow-up), setting**	**Sample size, age, setting, sample characteristics**	**Baseline cannabis, tobacco, mental health**	**Intervention(s) format and content, comparator**	**Outcome measure(s), analysis adjusted for**	**Intervention effect(s) at last follow-up (unless specified)**	**Risk of bias**
*Cannabis smoking outcomes*							
**Babor, 2004,** **USA [41]**	**RCT** (efficacy), 3 conditions, 4 waves (15 months), Clinical setting	*N* = 450 adults (aged 18+), 68.4% male, 69% white, 12% AA, 17.1% Hispanic	All used cannabis on at least 40 of the past 90 days (M = 82 days), all had DSM-IV cannabis dependence diagnosis	**Motivational intervention:** MET, two 1-hour in-person sessions one month apart, therapist-delivered. Includes personal feedback on cannabis use, problems, and attitudes; strategies to increase motivation to change behaviours **Comparators:** MET + CBT + case management; delayed treatment control (DTC)	Patterns of cannabis smoking over past 90-days, number of joints smoked on a typical day. Adjusted for baseline use	✓ Compared to DTC, the MET group showed greater reductions in days smoked, with a medium effect size (d = 0.59), and joints smoked per day, with a small effect size (d = 0.29). Reductions in days smoked in the MET condition were lesser than those from the MET + CBT + case management condition	Some concerns (RoB2)
**Bonar, 2022, USA [49]**	**RCT** (effectiveness/pilot), 2 conditions, 3 waves (6 months), Remote (online)	*N* = 149 young adults (aged 18–25, mean 21.0, SD 2.2), 55.7% female sex, 47.7% female gender, 42.3% male gender, 70.5% White, 20.1% AA, 9.4% Other, 20.1% Hispanic	All used cannabis in the past-month cannabis (≥3 times per week), mean past month use at 7.2, SD 1.1 (0 = never, 8 = more than once a day); smoking most common (I = 93%, C = 87%), followed by vaping, edibles, dabbing. Some cannabis measures were higher in the intervention versus control group. Overall, 42.3% met depression criteria, 45.0% for anxiety	**Social media intervention:** Participants joined a social media group managed by ‘e-coaches’ who post daily over 56 days to promote cannabis harm reduction. Topics: dealing with stress, what young adults do, strategies to stay safe while using, dealing with difficult situations, relationships with friends/family, reducing use, staying healthy, free time activities **Comparator:** Active control—social media group with content unrelated to substance use	Past 30-day use of cannabis across four modalities: smoking, vaping, dabbing, eating. Assessed using the Timeline Followback technique Adjusted for baseline use, age, sex, recreational cannabis legality	× There were no significant differences between intervention and control in total days smoked, times smoked, or quantity smoked at either follow-up. Both groups showed some reductions over time, with slightly larger decreases in total days smoked in the intervention group, but the effect sizes were small (d values ranged from −0.09 to 0.12 for T2 and −0.25 to 0.04 for T3)	Some concerns (RoB2)
**Bonsack, 2011, Switzerland [63]**	**RCT** (efficacy), 2 conditions, 4 waves (12 months), Clinical setting	*N* = 62 young adults (aged 18–35), 87.1% male	82.3% DSM-IV cannabis dependence, 27.3 mean joints per week All had a DSM-IV psychotic disorder diagnosis (66.1% schizophrenia)	**Motivational intervention**: MI, up to 9 psychologist-delivered sessions (30–60 minutes each: two individual sessions a week apart, then up to 4 individual and 3 group sessions up to 6 months in). Sessions aim to reduce harmful cannabis use and involve personalized feedback, discussions and fact sheets about cannabis and psychosis, pros and cons of changing cannabis use, and liaison with a clinician or case manager **Comparator:** TAU control	Number of joints smoked in the past week	✓ Joints smoked decreased significantly over time in both groups. This reduction was significantly larger in the MI group compared to the control at 3 months and 6 months, with a medium-to-large effect size (*d* = 0.65). By 12 months, the between-group difference was no longer significant (*d* = 0.37)	Some concerns (RoB2)
**Buckner, 2019, USA [48]**	**RCT** (effectiveness/pilot), 2 conditions, 2 waves (2 weeks), University setting	*N* = 63 young adults (mean age 18.9), 89.2% female, 89.2% non-Hispanic	All past-month cannabis use, 49.2% weekly cannabis use, 20.6% current social anxiety, 13.5% anxiety treatment history	**Personalized feedback:** single online session aiming to reduce cannabis use and manage negative affect. Personalized feedback, questions and feedback on knowledge about cannabis, exercises exploring motivation, reasons, and strategies to change cannabis use and manage negative affect **Comparator:** assessment only control	Cannabis use frequency, from the number of joints (i.e. ‘cannabis cigarettes’) per day over the past two weeks. Adjusted for baseline use and negative affect	✓ The intervention significantly reduced cannabis use frequency, particularly those with moderate to high social anxiety (*b* = −1.74, *P* < .05; *b* = −3.37, *P* < .01, respectively), but had no effect on those with low social anxiety. Further, the Condition × Social Anxiety interaction was significant (*b* = 0.11, *P* < .05)	Some concerns (RoB2)
**Clair, 2013, USA [43]**	**RCT** (efficacy), 2 conditions, 3 waves (3 months), Juvenile correction facility	*N* = 147 adolescents (aged 14–19), 85.7% male, 32.7% White, 34.7% Hispanic, 32.7% AA	89% had a past-year cannabis-related diagnosis. Mean (SD) number of joints on smoking days by ethnicity were: 7.59 (6.55) for AA, 6.64 (6.25) for White, 9.55 (9.85) for Hispanic.	**Motivational intervention**: MI, two in-person sessions delivered by research counsellors, aiming to reduce use and associated risky behaviours. MI sessions also included personalized feedback, goal setting, and handouts **Comparator:** Relaxation therapy	Average number of joints smoked on smoking days. Adjusted for baseline use	× There were no significant main effects or interaction effects of the intervention on cannabis outcomes	Some concerns (RoB2)
**Dupont, 2015, Netherlands [68]**	**Non randomized study** (effectiveness/pilot), 1 condition, 2 waves (1 week), Unspecified setting	*N* = 31 adolescents and young adults (aged 14–24), 71% male	All used cannabis in the past 30-days with signs of problematic use. Past week cannabis frequency mean = 4.3 (SD 4.2), daily tobacco cigarettes mean = 13.9 (SD 17.6)	**Mix of feedback, motivational, psychoeducational techniques:** 4 weekly in-person sessions delivered by a prevention worker. ‘Moti-4’ combines MI and personalized feedback with content on assessing use, use reasons and consequences, knowledge transfer, social network and peer pressure, plan for change and follow-up, optionally meeting with parents/educators	Number of joints smoked in the past week	✓ There was a significant decrease in past week number of joints smoked, with a medium to large effect size (*d* = 0.52)	Serious risk (ROBINS-I)
**Dupont, 2016, Netherlands [67]**	**RCT** (efficacy), 2 conditions, 3 waves (6 months), Clinical setting	*N* = 96 adolescents and young adults (ages 14–24, M = 18.0, SD 2.6), 84% male	Mean of 3.93 (SD 2.48) cannabis use session per week. Mean of 9.4 (SD 7.6) tobacco cigarettes per day. No differences between conditions	**C1: Mix of feedback, motivational, psychoeducational techniques:** 4 weekly in-person sessions delivered by a prevention worker. ‘Moti-4’ combines MI and personalized feedback with content on assessing use, use reasons and consequences, knowledge transfer, social network and peer pressure, plan for change and follow-up, optionally meeting with parents/educators. **C2: Psychoeducation** (presented as active control): 1 hour session discussing cannabis’ effects, including a post-session knowledge test and a take-home leaflet. Facilitators avoided using MI techniques	Number of joints smoked each week (mean). Adjusted for cannabis use, sex, educational level, norm	C1 ✓ | C2 × There was a significant reduction in weekly joints smoked between from baseline to the 6 month follow up (*b* = −4.05, SE = 1.95, *P* < .05), but no difference at the short-term post-test, in the C1 intervention group. This was not observed in the C2 intervention group	Some concerns (RoB2)
**Favrod, 2013, Switzerland [61]**	**Cohort** (efficacy/pilot), 1 condition, 4 waves (1 year), Clinical setting	N = 15 adults (median age 26.0, SD 15.0), 93.3% male	Median of 38.0 (SD 67.0) joints per week, 85.7% past month cannabis use days. All had DSM-IV diagnoses for psychotic disorders (73.3% schizophrenia), with 4.5 (SD 14) median years of treatment	**Motivational intervention**: 3 in-person group MI sessions (75 minutes) over 6 months, delivered in the context of roleplay sessions, including a debate on a cannabis-related theme, a trial of cannabis, and an ‘angel and demon’ cannabis temptation scenario. Sessions are steered by facilitators trained in MI techniques. Participants also received individual MI sessions (mean of 5.73 sessions, SD 2.68)	Number of joints over the past week, using the Timeline Followback technique	✓ Weekly joint consumption reduced from baseline to the 3- and 6-month follow-ups with large effect sizes (*r* = −0.60 and *r* = −0.59). This was maintained at one year with a moderate effect size (*r* = −0.37)	High risk (NOS Cohort)
**Fischer, 2012–2021, Canada [69] Fischer, 2012–2022, Canada [39]**	**RCT** (effectiveness), 4 conditions, 3 waves (3 and 12 months), Research lab	At 3 months, *N* = 113 young adults (aged 18–28, mean 20.6, 95% CI 2.3–2.9). 68.1% male, 74% White, 10% Arabic, 8% Asian, 8% Other. *N* = 76 at 12 months	All used cannabis for at least 1 year and at least 12 of the past 30 days. Mean cannabis use days = 23.79 (95% CI 22.82–24.77). Mean years of cannabis use at baseline =5.5 (SD 2.7)	**Mix of feedback, motivational, psychoeducational techniques:** Single individual sessions. Two versions of a brief intervention on cannabis health risks, advice to reduce risk, and brief motivational components: 1. In-person, psychologist-delivered (20–30 minutes); 2.8 page booklet (analysed combined) **Comparator:** a general health brief intervention (e.g. nutrition, stress) also offered in both oral and written modalities	Deep inhalation/breathholding techniques when using cannabis in the past 30 days (yes/no)	✓ The prevalence of deep inhalation/breathholding significantly decreased from baseline to 3 months in the total sample (79.7% to 63.7%, *P* < .001) and in cannabis intervention groups (e.g. combined BIs: 77.8% to 51.6%, *P* = .001), but not in the control health interventions. At follow-up, reductions remained significant for the cannabis BIs (*Q* = 13.1, *P* < .05) but not for controls. Effects were maintained at 12 months	Some concerns (RoB2)
**Gray, 2005, UK [65]**	**Non randomized study** (effectiveness/pilot), 2 conditions, 2 waves (3 months), Further education colleges	*N* = 140 adolescents (mean age intervention 17.0, SD1.0, control 17.7, SD 1.5), 53% female. Intervention: 48% White, 27% Black, 10% Asian, 15% Mixed/other (Control: 23%, 32%, 30%, and 15%, respectively)	Intervention: 52% currently used cannabis, 21% smoked cannabis weekly (control: 34% and 17%) Intervention: 98% ever smoked cigarettes, 58% smoked daily (Control: 87% and 60%)	**Motivational intervention**: Single individual in-person session (20–35 minutes), delivered by youth workers. MI to reduce cannabis, alcohol, and/or tobacco use **Comparator:** Assessment-only control	Past 30-day number of days of cannabis smoking, and past-week number of cannabis smoking occasions Adjusted for baseline variables that were non-equivalent between groups	× From baseline to follow-up, there was no significant change in cannabis smoking days in either group. Among those who used cannabis at baseline, past-week cannabis smoking occasions rose slightly in both groups, and mean number of days smoked in the past month also changed only slightly in both groups (Control: from 9.0 to 9.6 days; MI: 12.3 to 11.6)	Moderate risk (ROBINS-I)
**Laporte, 2017, France [70]**	**Cluster RCT** (effectiveness), 2 conditions, 4 waves (1 year), Clinical setting	N = 262 adolescents and young adults, (ages 15–25, mean 20.60, SD 2.6), 64.5% male	All used cannabis, 20 median joints per month (IQR 6–60), 46.2% used daily (≥30 joints per month). Past-month tobacco smoking was lower in the intervention group (87.9%) than control (95.7%, *P* = .02). 3.8% used psychotropic medication	**Mix of feedback, motivational, psychoeducational techniques:** CANABIC intervention, up to 4 sessions (mean 2.8) delivered by general practitioners (GPs). Content focused on reducing cannabis use through feedback, responsibility, advice, menu, empathy, self-efficacy **Comparator:** routine GP care control	Number of joints and bongs smoked per month Adjusted by age of first consumption, sex, CAST score, SES, and GP characteristics	×/✓ There were no overall significant differences in joints smoked between the intervention and control groups at any time point. Subgroup analyses revealed an effect for those who used cannabis nondaily at baseline, who smoked fewer joints than controls at 6 months (5 versus 10, *P* < .01) and 12 months (3 versus 10, *P* = .01). Those aged under 18 also smoked fewer joints than controls at 6 months (12.5 versus 20, *P* < .05)	Some concerns (RoB2)
**Lee, 2013, USA [57]**	**RCT** (efficacy), 2 conditions, 3 waves (6 months), University and remote	*N* = 212 young adults (aged 18–25, mean 20.0, SD 1.6). 45.3% female, 74.8% White, 10.5% Asian or PI, 14.7% Other, 5.7% Hispanic	All reported cannabis use for 5+ days in the past month. Mean (SD) past month days of cannabis use = 16.52(8.2) for the intervention group, 15.64(8.8) for control. Mean (SD) joints smoked per typical week = 9.35(9.8), for the intervention group, 8.29(9.5) for control	**Personalized feedback:** Single, hour-long, web-based session, adapted from the Teen Marijuana Check-Up, facilitated by trained research staff. Participants received personalized feedback on their cannabis use, using MI principles, with domains including: typical pattern of use, comparison to peers, social/academic/health/financial consequences, family history, pros and cons of change, other substance use, social networks, goals **Comparator:** assessment-only control	Typical number of joints smoked in a typical week during the past 60 days (adapted from the Daily Drinking Questionnaire)	×/✓ Participants in the intervention group reported a significantly lower number of joints smoked per week at 3 months compared to the control group (RR = 0.76 [0.60–0.96], *P* < .05), but this effect was not maintained at 6 months	Some concerns (RoB2)
**McCambridge, 2008, UK [66]**	**RCT** (effectiveness), 2 conditions, 3 waves (6 months), Further education colleges	*N* = 326 adolescents (aged 16 to 19, mean = 17.95). 69% male, 10.5% White, 52% Black, 19.5% Asian, 18% Mixed/other	Mean (SD): joints past week C1 = 10.3 (10.9), C2 = 11.1 (14.7); 30-day cannabis frequency C1 = 17.3 (9.8), C2 = 18.3 (10.4); GHQ-12 scores C1 = 11.2 (5.5) and C2 = 11.1 (5.7). Ever cigarette smoking 95% in C1 and 94% in C2	Both conditions were one hour-long session delivered by research staff. **C1. Motivational intervention:** MI on cannabis use, with secondary discussions on tobacco, alcohol, other drugs. Content on values and goals, risks, problems and concerns, decision-making, and self-monitoring or behaviour change. **C2. Psychoeducation:** ‘drug information and advice’ condition comprised leaflets with harm reduction information on alcohol and/or cannabis and/or tobacco; leaflets were discussed with facilitators, avoiding MI techniques	Joints smoked past week. Adjusted for practitioner, contamination risk, and sham saliva test	C1 ×/✓ | C2 ×/✓ Mean joints past week significantly reduced over time for the whole sample (10.3–11.1 at baseline; 4.6–15.9 at 3 months; 13.8–14.5 at 6 months); however, there were no significant differences between both intervention conditions	Some concerns (RoB2)
*Tobacco smoking outcomes*							
**Becker, 2014, Switzerland [62]**	**RCT** (efficacy), 3 conditions, 2 waves (8 weeks), Remote (online)	N = 261 adults who recently used tobacco and cannabis (mean age 29.8), 14.9% female in C1, 17.6% in C2, 30.3% in C3	2.4 mean cannabis use times per day, 12.9 mean cigarettes per day (there was no significant difference between conditions)	All three conditions involved one online session aiming to reduce tobacco use, cannabis use, and 'co-smoking' **C1: Personalized feedback** on tobacco and cannabis use and advice to reduce/quit. **C2: Motivational intervention:** MI with open-ended questions, affirmative feedback, and periodic summaries to enhance motivation to reduce/quit. **C3: Psychoeducation** with information about tobacco and cannabis and co-use (presented as an active control)	Daily frequency of tobacco use (typical daily number of cigarettes smoked × number of past month smoking days). Adjusted for gender and baseline readiness to quit cannabis	× There were no significant effects of time (baseline to 8 weeks), intervention condition, or time*intervention interaction on tobacco smoking outcomes	Some concerns (RoB2)
**Carpenter, 2024,** **USA [42]**	**RCT** (efficacy/pilot), 2 conditions, 2 waves (3 months), Remote (phone)	*N* = 72 adults (21+, mean age 51.7, SD 13.6), 50% female, 74% White, 16% Black, 8% Multiracial, 2% NA, 1% PI	Mean of 16.3 (SD 7.7) daily cigarettes, 24.8 (SD 7.1) past-month cannabis use days. 33% screened for depression (PHQ-2), 33% for anxiety (GAD-2)	**Mix of feedback, motivational, psychoeducational techniques:** 5 phone sessions delivered by trained coaches. Sessions include feedback on patterns of use, psychoeducation on cannabis and co-use, MET focusing on motivation to change and goals. Integrated with tobacco Quitline + NRT. **Comparator:** Tobacco Quitline + NRT only	Tobacco use: cigarettes per day and 7-day tobacco point prevalence abstinence	× There were no significant differences in tobacco use between the intervention and control conditions at follow-up, although tobacco use declined in both groups	Some concerns (RoB2)
**Dupont, 2015, Netherlands [68]**	**Non randomized study** (effectiveness/pilot), 1 condition, 2 waves (1 week), Unspecified setting	*N* = 31 adolescents and young adults (aged 14–24), 71% male	All used cannabis in the past-month, mean of 13.9 daily cigarettes	**Mix of feedback, motivational, psychoeducational techniques:** 4 weekly in-person sessions delivered by a prevention worker. ‘Moti-4’ combines MI and personalized feedback with content on assessing use, use reasons and consequences, knowledge transfer, social network and peer pressure, plan for change and follow-up, optionally meeting with parents/educators	Daily tobacco cigarettes smoked	× Daily cigarettes smoked did not significantly change from pre-test to post-test	Serious risk (ROBINS-I)
**Feldstein Ewing, 2013, USA [44]**	**Cohort** (efficacy), 1 condition, 2 waves (1 month), Various settings	*N* = 42 adolescents involved with a juvenile justice programme (aged 14 to 17, mean = 16.09, SD 1.09), 83.7% male, 53.5% Hispanic, 20.9% White, 14% Multiracial, 7% AA, 4.7% NA	All used cannabis, 97.7% used on weekdays 65.1% used tobacco >1 day in the past month	**Motivational intervention**: Two hour-long in-person sessions, delivered one week apart by a trained therapist **Comparator:** None	Tobacco use days, using the Timeline Followback technique	× Past-month tobacco use days did not change significantly from baseline to follow-up	High risk (NOS Cohort)
**Laporte, 2017, France [70]**	**Cluster RCT** (effectiveness), 2 conditions, 4 waves (1 year), Clinical setting	*N* = 262 adolescents and young adults, (ages 15–25, mean 20.60, SD 2.6), 64.5% male	All used cannabis, 20 median joints per month (IQR 6–60), 46.2% used daily (≥30 joints per month). Past-month tobacco smoking was lower in the intervention group (87.9%) than control (95.7%, *P* = .02). 3.8% used psychotropic medication	**Mix of feedback, motivational, psychoeducational techniques:** CANABIC intervention, up to 4 sessions (mean 2.8) delivered by general practitioners (GPs). Content focused on reducing cannabis use through feedback, responsibility, advice, menu, empathy, self-efficacy **Comparator:** routine GP care control	Quantity of cigarettes consumed. Adjusted by age of first consumption, sex, CAST score, SES, and GP characteristics	× The lower proportion of people who smoked tobacco at baseline in the intervention group compared to the control group persisted at 12 months. No other data is reported	Some concerns (RoB2)
**McCambridge, 2008, UK [66]**	**RCT** (efficacy), 2 conditions, 3 waves (6 months), Further education colleges	*N* = 326 adolescents (aged 16 to 19, mean = 17.95). 69% male, 10.5% White, 52% Black, 19.5% Asian, 18% Mixed/other	Mean (SD): joints past week C1 = 10.3 (10.9), C2 = 11.1 (14.7); 30-day cannabis frequency C1 = 17.3 (9.8), C2 = 18.3 (10.4); GHQ-12 scores C1 = 11.2 (5.5) and C2 = 11.1 (5.7). Ever cigarette smoking 95% in C1 and 94% in C2	Both conditions were one hour-long session delivered by research staff. **C1. Motivational intervention:** MI on cannabis use, with secondary discussions on tobacco, alcohol, other drugs. Content on values and goals, risks, problems and concerns, decision-making, and self-monitoring or behaviour change. **C2. Psychoeducation:** ‘drug information and advice’ condition comprised leaflets with harm reduction information on alcohol and/or cannabis and/or tobacco; leaflets were discussed with facilitators, avoiding MI techniques	Cigarette 30-day frequency, cigarettes per day Adjusted for practitioner, contamination risk, and bogus saliva test	C1 ×/✓ | C2 ×/✓ Mean 30-day cigarette frequency significantly decreased over time for the whole sample (19.5 at baseline; 18.1 at 3 months; 18.0 at 6 months); however, there were no significant differences between both intervention conditions	Some concerns (RoB2)

Symbols: ✓ significant reduction, × no significant reduction, and ×/✓ mixed findings.

AA, African American; CBT, cognitive behavioural therapy; GHQ-12, General Health Questionnaire 12 item; MET, motivational enhancement therapy; MI, motivational interviewing; NA, Native American; NRT, nicotine replacement therapy; RCT, randomized controlled trial; SD, standard deviation.

#### Intervention characteristics

Of 16 studies focused on uptake prevention, 14 evaluated school-based youth education programmes [37, 38, 45–47, 50–54, 58–60, 71, 72], and one delivered in youth clubs [55]. These were typically facilitated by teachers and delivered across multiple sessions. Six studies were based on the same curriculum, ‘keepin’ it REAL (kiR)’, which focuses on developing skills such as decision-making and peer resistance to prevent drug use [51, 53, 58–60, 72]. One study evaluated *DARE*, kiR’s precursor [47], two studies evaluated other school curricula, with similar emphasis on resistance skills [46, 71], and five studies were based on ‘Life Skills Training (LST)’, which incorporates refusal skills within broader life skills [37, 38, 45, 54, 55]. Additionally, two studies evaluated different uptake prevention interventions: one anti-substance-use and anti-cannabis television ads [56], another comparing motivational interviewing (MI; a goal-oriented approach for enhancing motivation to change behaviours) to ‘drug awareness’ psychoeducation (quiz, discussion, and leaflets) in further education [64].

Of 16 studies focused on promoting cessation and/or harm reduction, eight involved motivational interventions [41, 43, 44, 61–63, 65, 66] comprising MI or motivational enhancement therapy (MET; an adaptation of MI specific to substance use), typically in one to three sessions, except one lasting up to nine [63]. For motivational interventions, clinical settings were most common, most sessions were individual except for two studies [61, 63], and facilitators included psychologists, counsellors, research staff, and youth workers. Three studies assessed interventions delivering personalized feedback on drug use, each comprising a single hour-long online session [48, 57, 62]. Three studies assessed psychoeducation interventions about drug use [62, 66, 67] which were single-session, using leaflets and discussions without motivational techniques; one included a knowledge test [67]. Five studies assessed interventions combining motivational components with personalized feedback and psychoeducation; most were delivered in clinical/research settings over 1–4 sessions, facilitated by psychologists, general practitioners, and prevention workers [39, 67–70], and one was delivered remotely [42]. One social media intervention was led by ‘e-coaches’ posting daily cannabis harm reduction content for several weeks [49].

#### Outcome measures

All data were self-reported through questionnaires and/or interviews. Although four studies incorporated limited outcome bio-verification, these were not considered as outcome measures in this review, as they did not meet our criteria: two were sham bio-verifications to encourage accurate self-reporting [38, 54, 66], one was measured at baseline only [41], and another used tobacco bio-verification for a limited subset of participants only [42].

Interventions to prevent uptake most commonly included measures of past 30-day cannabis smoking, with other outcomes ranging from ever use [37, 38, 45, 54, 56] to past-week frequency [64]. Most measures referenced ‘cannabis smoking’ broadly, with one study referencing ‘number of puffs taken’ [72] and two measuring ‘joints’ smoked [59, 64]. One study included a cigarette smoking and cannabis co-use outcome [49].

Interventions to promote cessation or harm reduction typically included measures of cannabis quantity (e.g. number of joints smoked); one explored mode-specific use (e.g. smoking, vaping, dabbing, and eating) [49] and another assessed inhalation technique (e.g. deep inhalation or breath holding) [39, 69]. Most outcomes were measured over the past week or past month. Tobacco use was reported in six studies [42, 44, 62, 66, 68, 70] with outcomes including number of cigarettes smoked and frequency of smoking days.

### Effects of health education interventions aiming to prevent uptake

#### Cannabis smoking outcomes

There were mixed effects of the 15 studies evaluating uptake prevention interventions which reported cannabis smoking outcomes (Table 2). Five reported significant reductions in cannabis smoking, five showed partial effects (e.g. only among certain subgroups or time points), and five found no effect.

Three studies evaluating LST-based school programmes consistently demonstrated an effect on reducing cannabis smoking [37, 38, 45, 54, 55]. In contrast, seven studies assessing kir/*DARE* curriculums yielded varying results: two found significant reductions in cannabis smoking [51, 72], three found partial effects by gender (males only) [58, 59] or school level (secondary but not elementary school) [53], and three found no effects [47, 52, 60]. Two studies evaluating other youth education programmes, which also consisted of multiple school-based sessions aiming to increase knowledge of drug risks and build resistance skills, found no intervention effects on cannabis smoking [46, 71]. Televised advertisements were associated with reduced cannabis smoking at 12-months follow-up in one study, but only among people who used cannabis at baseline cannabis and responded positively to the ads [56]. While single-session MI and psychoeducation interventions both resulted in reductions in cannabis smoking at 6 and 12 months in one study, there were no significant differences between the two conditions [64].

#### Tobacco smoking and co-use outcomes

One study assessing health education interventions focused on preventing uptake reported a tobacco-related outcome (Table 3). This secondary school education programme showed an effect in reducing weekly cannabis and cigarette co-use in both general samples and a subsample of students who received at least 60% of the curriculum; monthly co-use declined only in this subsample [50].

### Effects of health education interventions that promote quitting or harm reduction

#### Cannabis smoking outcomes

Results are presented in Table 3. Effects of motivational interventions and combined motivational, feedback, and psychoeducation interventions on reducing cannabis smoking were promising. Six studies reported a significant impact on cannabis smoking outcomes, while another found an overall reduction in cannabis smoking but with no significant difference between a motivational intervention and psychoeducation intervention [66]. Another study observed a reduction in cannabis smoking only among specific subgroups: those with baseline non-daily use and those under 18 years old [70]. However, two studies evaluating motivational interviewing among adolescents found no effect on cannabis smoking outcomes compared to relaxation therapy and assessment-only controls [43, 65].

Compared to assessment-only controls, the two studies evaluating personalized feedback interventions demonstrated a reduction in cannabis smoking [48, 57], although one did not find a sustained effect at 6-months follow-up [57]. Findings for the two psychoeducation interventions were mixed; one study found a reduction in joints smoked, but no greater than the motivational intervention comparator [66], and one found no effect on joint consumption [67]. A social media intervention did not reduce cannabis smoking compared to an active control group using content unrelated to substance use [49]; however, this was the only study assessing different consumption modalities, and it found an effect in reducing days of cannabis vaping at 6 months follow-up.

#### Tobacco smoking and co-use outcomes

Six studies assessing health education interventions promoting cessation and/or harm reduction reported tobacco smoking and co-use outcomes among people who use cannabis (Table 3) [42, 44, 62, 66, 68, 70]. Results for motivational interventions were not promising: two found no effects on tobacco smoking at follow-up, neither over time [44, 62] nor compared to feedback or psychoeducation interventions [62], and one was associated with a reduction in cigarette smoking; however, this was not different from the comparator condition [66]. Similarly, of the three studies evaluating interventions combining feedback, motivational, and psychoeducational techniques [42, 68, 70], only one was associated with a reduction in cigarette smoking and this reduction did not differ from the control condition [42]. For psychoeducation interventions, no effect was observed [62] or an effect over time which was no different from the motivational comparator condition [66].

Only two studies reported mental health outcomes; these findings are summarized in Appendix D.

### Risk of bias in studies

Risk of bias was high overall; it is reported in Tables 2 and 3, with further details in Appendix E and OSF [35]. All but one of the 25 RCTs had moderate risk of bias, and the remaining one high risk; this was mainly due to lack of participant and assessor blinding and unregistered analyses. High attrition rates were common but addressed using various methods. Of four non-randomized studies, two had serious risk of bias and two moderate, mainly due to self-report limitations and unregistered analyses. Two lacked key details on confounder adjustments, and two may have been biased by attrition. Among three cohort studies, two had high risk of bias due to lack of confounder adjustments, limited generalizability, and reliance on self-report measures.

## Discussion

The reviewed health education intervention evaluations, comprising both efficacy trials conducted under controlled conditions and effectiveness trials in real-world settings, yielded mixed results in cannabis smoking prevention and reduction.

Among interventions aimed at preventing cannabis uptake, most studies evaluated universal prevention curricula delivered in secondary schools, predominantly in the US or adapted from US-developed ‘kiR or DARE or Life Skills Training (LST)’ programmes. LST was consistently effective in preventing and reducing cannabis smoking but was assessed only by two US-based research teams and required significant resources. Differences in regulation, social norms, and educational systems may affect outcomes and hence generalizability of the findings to other countries and contexts (e.g. Europe, where cannabis laws, perceptions, and use patterns differ [1, 10]). Scalability should also be considered, as it varies across interventions: school-based or online programmes may be easier to implement broadly than resource-intensive approaches such as motivational interventions. Motivational interviewing and psychoeducation showed some promise in reducing cannabis smoking, based on a single UK RCT.

Interventions to promote cessation and harm reduction also yielded mixed results across smaller, often pilot studies with diverse populations, settings, and formats. Motivational and combined approaches (integrating motivation, personalized feedback, and psychoeducation) showed promise, with 6 studies reporting cannabis smoking reductions, though effects were inconsistent across subgroups and not always sustained. Two personalized feedback interventions also showed promise in reducing cannabis smoking, psychoeducation findings were mixed, and a social media-based intervention had no effect on cannabis smoking but reduced cannabis vaping.

For tobacco outcomes, more limited evidence from seven studies suggests that health education interventions were less effective in reducing tobacco smoking among people who use cannabis than in reducing cannabis smoking. Motivational interventions, personalized feedback, and psychoeducation showed little effect on tobacco smoking. Only two studies reported tobacco smoking reductions over time, one assessing both a motivational intervention and psychoeducation among UK adolescents, the other a combined intervention (motivation, feedback, and psychoeducation) among US adults, but these reductions did not differ from comparator conditions. Heterogeneous interventions and limited information on patterns of co-use constrain interpretation of why these programmes were not effective in reducing tobacco smoking among people who use cannabis. A single RCT indicated that school-based universal prevention curricula might reduce both cannabis and tobacco co-use, highlighting the need for further research given the high prevalence of co-use.

Only two studies examined mental health outcomes. Although those two interventions successfully reduced cannabis smoking at follow-up, no changes in depression, anxiety, or psychotic disorder symptoms were found. As these studies did not report tobacco smoking outcomes, it is possible that lack of effect on mental health outcomes was partly due to continued tobacco use. This highlights the need for greater focus on mental health and tobacco co-use when designing and evaluating health education interventions to address cannabis smoking.

### Quality of evidence and methodology

Risk of bias was high in most studies and methodological issues were common, thus limiting the conclusions that can be made from this review. Bias was introduced due to lack of pre-registration (not yet a standard practice at the time of publication for some included studies), high attrition, reliance on self-reported outcomes, and issues with assessor and participant blinding. Strategies exist to mitigate these issues, such as ensuring more comparable control conditions to improve participant blinding in RCTs [73]. Further, many cessation and harm reduction interventions studies involved a majority of male participants, which may not be representative in all contexts—for example, women make up close to half of people who use cannabis in North America [1]. Race/ethnicity was reported inconsistently and omitted in 11 studies, limiting assessment of impacts across groups.

Importantly, few studies considered how cannabis was consumed—with only one study reporting outcomes for smoking, vaping, edibles, and dabbing—often assuming smoking as the mode of administration. Despite the documented prevalence of co-use [9–11], many studies omitted tobacco smoking outcomes among people who use cannabis. Inconsistent measurement of cannabis–tobacco co-use has been identified as a barrier to fully understanding its public health impact and developing effective strategies to address it [11]. Researchers have described the challenges in measuring cannabis use including quantity, frequency, and mode of use [74]; the National Academies of Sciences, Engineering, and Medicine recommends the development of standardized terminology and evidence-based question banks to improve cannabis research [3]. Moreover, most interventions did not explicitly target smoking-related harms or address co-use, which may have limited their impact in reducing smoking-specific health risks.

### Limitations and strengths

Some limitations should be noted. First, the smoking outcomes criteria may have excluded studies that measured cannabis smoking without explicitly reporting it as such, meaning some relevant health education interventions may not have been captured. Second, risk of bias assessments were conducted by a single researcher, and some reports were single-screened, though agreement was high among those that were, and inconsistencies easily resolved. Third, while the distinction between uptake prevention and cessation/harm reduction interventions was useful for organizing findings, some uptake prevention studies targeted older adolescents and young adults with substantial baseline cannabis use, and some cessation/harm reduction interventions also had uptake prevention aims. Nonetheless, this was the first review to systematically evaluate the impact of health education interventions on cannabis smoking and tobacco smoking among people who use cannabis, encompassing a broad range of intervention types, study designs, populations, and publication dates.

## Conclusion

Evidence for school-based education programmes to prevent cannabis smoking uptake is mixed, with some curricula and delivery contexts showing preventive effects, but many studies reporting no significant differences compared to control. Motivational interventions showed more consistent support for cannabis smoking cessation/reduction, although effects often diminished over time. Given the widespread use of cannabis and associated smoking-related harms, even modest, context-specific effects could have meaningful public health benefits. Future research should aim to clarify which programme components, delivery settings, and participant characteristics are associated with sustained reductions or prevention of smoking; it also should address different cannabis administration routes and co-use with tobacco/nicotine products using standardized terminology and measures.

## Supplementary Material

SysRev_Supplementary_Revised_cyag016

## Data Availability

The data underlying this article are available on the Open Science Framework (OSF) at: https://osf.io/azsh2/files/osfstorage.
