# Supplementary material for: Health education interventions to reduce cannabis and tobacco smoking-related harms among people who use cannabis: a systematic review
Source: Health Educ Res. 2026 May 21;41(3):cyag016. doi: 10.1093/her/cyag016 (PMC13192474; doi:10.1093/her/cyag016)
Supplement: SysRev_Supplementary_Revised_cyag016 [file sysrev_supplementary_revised_cyag016.doc]

# Supplementary materials *Health education interventions to reduce cannabis and tobacco smoking-related harms among people who use cannabis: a systematic review*

## Appendix A. PRISMA 2020 checklist

| **Section and Topic** | **Item #** | **Checklist item** | **Location where item is reported** |
| --- | --- | --- | --- |
| **TITLE** | | |  |
| Title | 1 | Identify the report as a systematic review. | Title |
| **ABSTRACT** | | |  |
| Abstract | 2 | See the PRISMA 2020 for Abstracts checklist. | Abstract |
| **INTRODUCTION** | | |  |
| Rationale | 3 | Describe the rationale for the review in the context of existing knowledge. | Introduction |
| Objectives | 4 | Provide an explicit statement of the objective(s) or question(s) the review addresses. | Introduction |
| **METHODS** | | |  |
| Eligibility criteria | 5 | Specify the inclusion and exclusion criteria for the review and how studies were grouped for the syntheses. | Methods, Supplementary |
| Information sources | 6 | Specify all databases, registers, websites, organisations, reference lists and other sources searched or consulted to identify studies. Specify the date when each source was last searched or consulted. | Methods, Supplementary |
| Search strategy | 7 | Present the full search strategies for all databases, registers and websites, including any filters and limits used. | Supplementary |
| Selection process | 8 | Specify the methods used to decide whether a study met the inclusion criteria of the review, including how many reviewers screened each record and each report retrieved, whether they worked independently, and if applicable, details of automation tools used in the process. | Methods |
| Data collection process | 9 | Specify the methods used to collect data from reports, including how many reviewers collected data from each report, whether they worked independently, any processes for obtaining or confirming data from study investigators, and if applicable, details of automation tools used in the process. | Methods |
| Data items | 10a | List and define all outcomes for which data were sought. Specify whether all results that were compatible with each outcome domain in each study were sought (e.g. for all measures, time points, analyses), and if not, the methods used to decide which results to collect. | Methods, Supplementary |
|  | 10b | List and define all other variables for which data were sought (e.g. participant and intervention characteristics, funding sources). Describe any assumptions made about any missing or unclear information. | Methods, Supplementary |
| Study risk of bias assessment | 11 | Specify the methods used to assess risk of bias in the included studies, including details of the tool(s) used, how many reviewers assessed each study and whether they worked independently, and if applicable, details of automation tools used in the process. | Methods |
| Effect measures | 12 | Specify for each outcome the effect measure(s) (e.g. risk ratio, mean difference) used in the synthesis or presentation of results. | Methods |
| Synthesis methods | 13a | Describe the processes used to decide which studies were eligible for each synthesis (e.g. tabulating the study intervention characteristics and comparing against the planned groups for each synthesis (item #5)). | Methods |
|  | 13b | Describe any methods required to prepare the data for presentation or synthesis, such as handling of missing summary statistics, or data conversions. | Methods |
|  | 13c | Describe any methods used to tabulate or visually display results of individual studies and syntheses. | Methods |
|  | 13d | Describe any methods used to synthesize results and provide a rationale for the choice(s). If meta-analysis was performed, describe the model(s), method(s) to identify the presence and extent of statistical heterogeneity, and software package(s) used. | Methods |
|  | 13e | Describe any methods used to explore possible causes of heterogeneity among study results (e.g. subgroup analysis, meta-regression). | NA |
|  | 13f | Describe any sensitivity analyses conducted to assess robustness of the synthesized results. | NA |
| Reporting bias assessment | 14 | Describe any methods used to assess risk of bias due to missing results in a synthesis (arising from reporting biases). | Methods |
| Certainty assessment | 15 | Describe any methods used to assess certainty (or confidence) in the body of evidence for an outcome. | NA |
| **RESULTS** | | |  |
| Study selection | 16a | Describe the results of the search and selection process, from the number of records identified in the search to the number of studies included in the review, ideally using a flow diagram. | Results |
|  | 16b | Cite studies that might appear to meet the inclusion criteria, but which were excluded, and explain why they were excluded. | NA |
| Study characteristics | 17 | Cite each included study and present its characteristics. | Results |
| Risk of bias in studies | 18 | Present assessments of risk of bias for each included study. | Results, Supplementary |
| Results of individual studies | 19 | For all outcomes, present, for each study: (a) summary statistics for each group (where appropriate) and (b) an effect estimate and its precision (e.g. confidence/credible interval), ideally using structured tables or plots. | Results |
| Results of syntheses | 20a | For each synthesis, briefly summarise the characteristics and risk of bias among contributing studies. | Results |
|  | 20b | Present results of all statistical syntheses conducted. If meta-analysis was done, present for each the summary estimate and its precision (e.g. confidence/credible interval) and measures of statistical heterogeneity. If comparing groups, describe the direction of the effect. | NA |
|  | 20c | Present results of all investigations of possible causes of heterogeneity among study results. | NA |
|  | 20d | Present results of all sensitivity analyses conducted to assess the robustness of the synthesized results. | NA |
| Reporting biases | 21 | Present assessments of risk of bias due to missing results (arising from reporting biases) for each synthesis assessed. | NA |
| Certainty of evidence | 22 | Present assessments of certainty (or confidence) in the body of evidence for each outcome assessed. | NA |
| **DISCUSSION** | | |  |
| Discussion | 23a | Provide a general interpretation of the results in the context of other evidence. | Discussion |
|  | 23b | Discuss any limitations of the evidence included in the review. | Discussion |
|  | 23c | Discuss any limitations of the review processes used. | Discussion |
|  | 23d | Discuss implications of the results for practice, policy, and future research. | Discussion |
| **OTHER INFORMATION** | | |  |
| Registration and protocol | 24a | Provide registration information for the review, including register name and registration number, or state that the review was not registered. | Methods |
|  | 24b | Indicate where the review protocol can be accessed, or state that a protocol was not prepared. | Methods |
|  | 24c | Describe and explain any amendments to information provided at registration or in the protocol. | Methods, Supplementary |
| Support | 25 | Describe sources of financial or non-financial support for the review, and the role of the funders or sponsors in the review. | Title page |
| Competing interests | 26 | Declare any competing interests of review authors. | Title page |
| Availability of data, code and other materials | 27 | Report which of the following are publicly available and where they can be found: template data collection forms; data extracted from included studies; data used for all analyses; analytic code; any other materials used in the review. | Methods, Results, Supplementary |

## Appendix B: Search Strategies, Eligibility Criteria, and Protocol Deviations

Initial searches were ran on 20 February 2024, and updated searches on 14 February 2025.

**Scopus database**:

- Searched as a document search (title, abstract, keyword) filtering by ‘article’
- Original query: ((smok* OR combust* OR inhal* OR burn* OR tobacco OR cigarette OR ((mode OR route OR modalit*) AND (administration OR consumption))) AND (intervention OR education OR literacy OR communication OR ((harm OR risk) AND reduction) OR campaign OR prevent* OR messag* OR curriculum OR guidelines OR media OR warning) AND cannabis OR marijuana OR marihuana OR hemp OR hashish OR bhang OR Ganja)
- Updated search query: ((smok* OR combust* OR inhal* OR burn* OR tobacco OR cigarette OR ((mode OR route OR modalit*) AND (administration OR consumption))) AND (intervention OR education OR literacy OR communication OR ((harm OR risk) AND reduction) OR campaign OR prevent* OR messag* OR curriculum OR guidelines OR media OR warning) AND cannabis OR marijuana OR marihuana OR hemp OR hashish OR bhang OR Ganja) AND ORIG-LOAD-DATE > 20240220
- 7,511 results in the initial search, 691 results in the updated search.

**Embase classic + Embase (Ovid) database:**

- Filtered by ‘article’
- Query (**#5** used for the updated search only):
  - **#1** (smok* or combust* or inhal* or burn* or tobacco or cigarette or ((mode or route or modalit*) and (administration or consumption))).mp
  - **#2** (Intervention or Education or Literacy or Communication or ((Harm or risk) and reduction) or Campaign or Prevent* or Messag* or Curriculum or Guidelines or Media or Warning).mp
  - **#3** cannabis.mp. or exp cannabis smoking/ or exp cannabis/ or exp "cannabis use"/
  - **#4** 1 AND 2 AND 3
  - **#5** limit 4 to dc=20240221-20250214
- 7,606 results in the initial search, 676 results in the updated search.

**Medline (Ovid) database:**

- Query (**#5** used for the updated search only):
  - **#1** (smok* or combust* or inhal* or burn* or tobacco or cigarette or ((mode or route or modalit*) and (administration or consumption))).mp.
  - **#2** (Intervention or Education or Literacy or Communication or ((Harm or risk) and reduction) or Campaign or Prevent* or Messag* or Curriculum or Guidelines or Media or Warning).mp.
  - **#3** cannabis.mp. or exp cannabis/
  - **#4** 1 AND 2 AND 3
  - **#5** 4 and (2024* or 2025*).ed.
- 2,724 results in the initial search, 278 results in the updated search.

**CINAHL (EBSCO) database:**

- Original search using the ‘boolean/phrases’ mode across default search fields (title, abstract, subject headings), unchecking ‘suggest subject terms’ and filtering by ‘academic journals’, using the query:
  - **#1** (cannabis OR marijuana OR marihuana OR hemp OR hashish OR bhang OR ganja)
  - **#2** ((Intervention or Education or Literacy or Communication or ((Harm or risk) and reduction) or Campaign or Prevent* or Messag* or Curriculum or Guidelines or Media or Warning))
  - **#3** ((smok* or combust* or inhal* or burn* or tobacco or cigarette or ((mode or route or modalit*) and (administration or consumption))))
  - **#4** 1 AND 2 AND 3
- Updated search using ‘Proximity’ mode, filtered for ‘Peer reviewed’ and publication date ‘February 2024’, using the query below. **#1**, **#2**, and **#3** were each run as abstract OR title.
  - **#1** (cannabis OR marijuana OR marihuana OR hemp OR hashish OR bhang OR ganja)
  - **#2** ((Intervention or Education or Literacy or Communication or ((Harm or risk) and reduction) or Campaign or Prevent* or Messag* or Curriculum or Guidelines or Media or Warning))
  - **#3** ((smok* or combust* or inhal* or burn* or tobacco or cigarette or ((mode or route or modalit*) and (administration or consumption))))
  - **#4** 1 AND 2 AND 3
- 3,090 results in the initial search, 114 results in the updated search.

**IBSS (ProQuest) database:**

- Searched filtering for ‘peer reviewed’ and ‘NOFT’ using the query below; updated searches with filter “2024-02-21 - 2025-02-17”.
  - noft((smok* or combust* or inhal* or burn* or tobacco or cigarette or ((mode or route or modalit*) and (administration or consumption))))
  - AND noft((Intervention or Education or Literacy or Communication or ((Harm or risk) and reduction) or Campaign or Prevent* or Messag* or Curriculum or Guidelines or Media or Warning))
  - AND noft(cannabis OR marijuana OR marihuana OR hemp OR hashish OR bhang OR Ganja)
- 509 results in the initial search, 8 results in the updated search.

**Eligibility criteria (PICOS):**

*Population*. Human participants of any age, without demographic restrictions.

*Intervention*. Health education interventions aimed at reducing health harms from smoking cannabis and/or the co-use of cannabis with combustible tobacco were included. Interventions could be delivered across populations, groups, jurisdictions, institutions, or at the individual level. They could include written and/or verbal content, be delivered through various media, and could constitute a subset of a wider intervention. Interventions restricting marketing or advertising content and extensive individualised psychosocial interventions beyond brief educational/motivational interventions (e.g., cognitive-behavioural therapy, mindfulness) were excluded.

*Comparison*. Any comparator or control used in the original studies.

*Outcomes*. The primary outcome was smoking behaviour, including cannabis use through all smoking routes of administration, and/or use of combustible tobacco (e.g., cigarettes, cigars, waterpipe), among people who use cannabis. Smoking behaviour could be measured as quitting/abstinence, changes in quantity or frequency of use, and/or switching from smoking to non-smoking routes of administration. The secondary outcomes were physical and mental health outcomes (e.g., respiratory health, cardiovascular health, cancers, overall morbidity and mortality, health care utilisation, quality of life, common mental health conditions), if reported in studies which measured the primary outcome.

*Study design*. Peer-reviewed reports using quantitative methods, including experimental and quasi-experimental study designs and analytical observational studies, were eligible; reports were not eligible if they were reviews or only reported data that were qualitative or case studies.

**Deviations from protocol**

Some eligibility criteria were refined for consistent screening by:

1. Specifying that tobacco smoking behaviour was included only if reported within a cannabis-using sample;
2. Excluding reports where the cannabis use measure did not explicitly mention cannabis *smoking*, and where no description or reference (e.g., to a validated instrument that could be checked) was provided to confirm that cannabis *smoking* was assessed;
3. Contacting authors for clarification when the Methods section lacked a clear cannabis use measure but administration methods were mentioned elsewhere (e.g., 'cannabis smoking,' 'joints'), with exclusion if no response was received.

Studies which measured numbers of joints consumed were only included if there was also a reference to joint *smoking* specifically elsewhere in the report, as joints may be used as a standardised measure of quantity of cannabis consumed through various modes.

Additionally, for risk of bias assessments, we used the NOS Cohort tool instead of the JBI Critical Appraisal Checklist for Cohort Studies, as the NOS provides a risk-of-bias rating, whereas the JBI tool primarily serves as a critical appraisal checklist to guide inclusion/exclusion decisions.

## Appendix C: Data extraction items

The completed data extraction form is available on OSF: <https://osf.io/azsh2/>.

| **Study metadata** | 1. Author, year 2. Title 3. Country where conducted 4. Funding source(s) 5. Declared COI |
| --- | --- |
| **Overview** | 1. Study aims and/or research questions 2. Synopsis (optional) 3. Study design – Conditions 4. Study design – Waves 5. Method(s) of data collection 6. Any related included study 7. Any other notes |
| **Sample characteristics** | 1. Sampling procedure 2. Inclusion/exclusion criteria for participants 3. Number of participants overall 4. Number of participants across conditions 5. Participants’ age (age group, age range, frequency statistics) 6. Participants’ gender or sex (frequency statistics) 7. Participants’ race / ethnicity (frequency statistics) 8. Other socio-demographics (frequency statistics) 9. Cannabis use characteristics at baseline 10. Substance use (other than cannabis) characteristics at baseline 11. Participants baseline physical and/or mental health characteristics |
| **Intervention characteristics** | 1. Health education intervention(s) name(s) 2. Comparison condition(s) 3. Health education intervention(s) aims / main focus 4. Cannabis and substance use components 5. Intervention(s) setting 6. Intervention(s) format / mode 7. Intervention(s) facilitator 8. Intervention(s) sessions and frequency 9. Intervention dates 10. Rates of completion, adherence, fidelity 11. Any other intervention info |
| **Outcome measures** | 1. Cannabis smoking measure(s) 2. Cannabis other mode of use measure(s) 3. Tobacco/nicotine measure(s) 4. Is tobacco/nicotine use measured among cannabis users specifically? (Yes/No) 5. Mental health measure(s) 6. Other relevant measure(s), e.g. general health |
| **Outcome results** | 1. Relevant analytical strategies 2. Within or between subjects? (Within/Between/Both) 3. Cannabis smoking results 4. Cannabis other modes of use results 5. Tobacco/nicotine results, if among cannabis users only 6. Mental health results 7. Other health results 8. Effective? (Yes/No for cannabis, tobacco, or both) |

## Appendix D. Mental health outcome findings

Of the 32 included studies, two also assessed mental and physical health outcomes at follow-up (Table III). Both were evaluations of motivational interventions, and both found reductions in cannabis smoking at follow-up, although neither had an effect on mental health outcomes [41, 45].

One examined 120 adults diagnosed with cannabis dependence; participants reported no significant changes in depression, anxiety, or dependence outcomes (measured using the Beck Depression Inventory, State portion of the State–Trait Anxiety Inventory, and the psychiatric component of the Addiction Severity Index) at the 9-month follow-up [41].

The other investigated 30 young adults (aged 18–35) with schizophrenia or another psychotic disorder who reported smoking ≥3 joints per week in the past month; no statistically significant differences were found at 12-months follow-up in psychotic disorders (measured using the Positive and Negative Syndrome Scale) or days in hospital [45].

## Appendix E. Risk of bias assessments

The completed risk of bias assessment forms are available on OSF: <https://osf.io/azsh2/>.

**Table E1.** Risk of bias assessment for randomised studies (n=25).

| **Cochrane risk-of-bias tool for randomized trials (RoB 2)** | **Domain 1: Randomization process** | **Domain 2: Deviations from the intended interventions** | **Domain 3: Missing outcome data** | **Domain 4: Measurement of the outcome** | **Domain 5: Selection of the reported result** | **Overall risk of bias**  *(Low Risk, Some Concerns, High Risk)* |
| --- | --- | --- | --- | --- | --- | --- |
| Ayers et al., 2023 | Low Risk | Some concerns | Low Risk | Some concerns | Some concerns | **Some concerns** |
| Carpenter et al., 2024 | Low Risk | Some concerns | Low Risk | Some concerns | Low Risk | **Some concerns** |
| Clayton et al., 1996 | Some concerns | Some concerns | Some concerns | Some concerns | Some concerns | **Some concerns** |
| Khuzwayo et al., 2020 | Some concerns | Some concerns | Low Risk | Some concerns | Some concerns | **Some concerns** |
| Kulis et al., 2021 | Some concerns | Some concerns | Low Risk | Some concerns | Low Risk | **Some concerns** |
| Kulis et al., 2007 | Low Risk | Some concerns | Low Risk | Some concerns | Some concerns | **Some concerns** |
| Marsiglia et al., 2011 | Some concerns | Some concerns | Low Risk | Some concerns | Some concerns | **Some concerns** |
| Marsiglia et al., 2015 | Some concerns | Some concerns | Low Risk | Some concerns | Some concerns | **Some concerns** |
| Spoth et al., 2007, 2011 | Low Risk | Some concerns | Low Risk | Some concerns | Some concerns | **Some concerns** |
| Spoth et al., 2002, 2008 | Low Risk | Some concerns | Low Risk | Some concerns | Some concerns | **Some concerns** |
| McCambridge et al., 2008 | Low Risk | Some concerns | Low Risk | Low Risk | Some concerns | **Some concerns** |
| McCambridge et al., 2011 | Low Risk | Some concerns | Low Risk | Some concerns | Some concerns | **Some concerns** |
| Laporte et al., 2017 | Some concerns | Some concerns | Low Risk | Some concerns | Low Risk | **Some concerns** |
| Marsiglia et al., 2018 | Some concerns | Some concerns | Some concerns | Some concerns | Some concerns | **Some concerns** |
| Okamoto et al., 2019 | Some concerns | High risk | High risk | Some concerns | Some concerns | **High risk** |
| Babor et al., 2004 | Some concerns | Some concerns | Low Risk | Some concerns | Some concerns | **Some concerns** |
| Becker et al., 2014 | Some concerns | Some concerns | Low Risk | Low Risk | Low Risk | **Some concerns** |
| Bonsack et al., 2011 | Low Risk | Some concerns | Low Risk | Some concerns | Some concerns | **Some concerns** |
| Botvin et al., 1995 | Some concerns | Some concerns | Some concerns | Some concerns | Some concerns | **Some concerns** |
| Clair et al., 2013 | Low Risk | Some concerns | Low Risk | Some concerns | Some concerns | **Some concerns** |
| Dupont et al., 2016 | Some concerns | Some concerns | Some concerns | Some concerns | Some concerns | **Some concerns** |
| Fischer et al., 2012-1, 2012-2 | Low Risk | Some concerns | Some concerns | Some concerns | Some concerns | **Some concerns** |
| Lee et al., 2013 | Low Risk | Some concerns | Low Risk | Low Risk | Some concerns | **Some concerns** |
| Bonar et al., 2022 | Some concerns | Some concerns | Low Risk | Low Risk | Low Risk | **Some concerns** |
| Bucker et al., 2019 | Low Risk | Some concerns | Low Risk | Low Risk | Some concerns | **Some concerns** |

**Table E2.** Risk of bias assessment for non-randomised (n=4) and cohort studies (n=3).

| **Risk Of Bias In Non-randomized Studies – of Interventions, Version 2 (ROBINS-I)** | **Confounding** | **Classification** | **Selection** | | **Deviations** | **Missing data** | | **Outcome measurements** | **Selection of reported results** | **Overall RoB**  *(Low to Critical)* |
| --- | --- | --- | --- | --- | --- | --- | --- | --- | --- | --- |
| Dupont et al., 2015 | Moderate | Low | Low | | Low | Low | | Moderate | Moderate | Serious Risk |
| Hecht et al., 2006 | Low | Low | Low | | Moderate | Moderate | | Moderate | Moderate | Serious Risk |
| Gray et al., 2005 | Low | Low | Low | | Low | Low | | Moderate | Moderate | Moderate Risk |
| St Pierre et al., 1992 | Moderate | Low | Low | | Moderate | Moderate | | Moderate | Moderate | Serious Risk |
|  |  |  |  | |  |  | |  |  |  |
| **Newcastle - Ottawa Quality Assessment Scale: Cohort Studies (NOS Cohort)** | **Selection** | | | **Comparability** | | | **Outcomes** | | | **Overall RoB**  *(Low to High)* |
| Alvaro et al., 2013 | 4 stars | | | 1 star | | | 2 stars | | | Low Risk (Good) |
| Favrod et al., 2013 | 2 stars | | | No stars | | | 2 stars | | | High Risk (Poor) |
| Feldstein Ewing et al., 2013 | 2 stars | | | No stars | | | 2 stars | | | High Risk (Poor) |
